# Medical Malpractice in the Waiting Room: Who Is at Risk?

**DOI:** 10.5811/cpcem.42039

**Published:** 2025-09-10

**Authors:** Kayla P. Carpenter, Laura Walker, Rachel A. Lindor

**Affiliations:** Mayo Clinic, Department of Emergency Medicine, Rochester, Minnesota

**Keywords:** malpractice, waiting room, EMTALA, negligence

## Abstract

**Introduction:**

Prolonged emergency department (ED) wait times pose problems for both patients and ED staff. Poor patient outcomes can result in litigation that could have been prevented by faster access to care.

**Case Series:**

We present 10 lawsuits involving patients who experienced poor outcomes allegedly due to inappropriate management in the waiting room. These cases involved allegations of violations of the Emergency Medical Treatment and Labor Act (EMTALA) or general negligence and were levied against both the physicians and hospitals involved.

**Conclusion:**

Both common law and EMTALA’s medical screening exam requirements impose significant obligations on physicians and hospitals to proactively manage patients in the waiting room. Being familiar with these requirements may help minimize legal risks.

## INTRODUCTION

A 50-year-old male experiencing indigestion and lightheadedness presented to an emergency department (ED) in North Carolina. After a prolonged delay and before receiving any evaluation, the patient left, suffering a fatal cardiac arrest moments later outside the hospital. The patient’s family sued, claiming that his death could have been avoided with faster medical screening. The hospital argued that the patient’s decision to leave was the cause of his death but ultimately settled with his family for $650,000.^1^

Although this case occurred more than 20 years ago, ED wait times have not improved during that period—a problem exacerbated by patient boarding, inadequate staffing, and increases in non-urgent visits.^2^ Prolonged wait times pose problems for patient satisfaction, staff satisfaction and perhaps, most importantly, patient safety.^3^,^4^ When patients experience poor outcomes that could have been prevented by faster access to care, they may choose to sue. Here, we will examine the legal obligations owed to patients in waiting rooms and how EDs can best attempt to meet these obligations. The two main avenues that pose legal risk are allegations of Emergency Medical Treatment and Labor Act (EMTALA) violation, or general negligence.

## LEGAL RISKS UNDER EMTALA

Since its enactment in 1986, EMTALA requires all hospitals that accept Medicare payments to provide a screening exam to patients seeking emergency care and stabilize any emergency medical conditions identified prior to patient discharge or transfer. While these requirements may seem straightforward, the majority of reported EMTALA lawsuits involving waiting room patients revolve around nuances of the screening requirement.

## EMTALA’S MEDICAL SCREENING EXAM REQUIREMENT

The medical screening exam (MSE) required by EMTALA must be offered to all patients seeking emergency care, and it must be timely and be designed to identify an emergency medical condition. The Centers for Medicare & Medicaid Services (CMS) does not delineate precise requirements for an MSE, but it requires that it be commensurate with the clinical conditions of the patient and be provided equally to all patients with that condition at that facility. So, patients with a sore throat do not need an MSE that is as extensive as patients with chest pain, but all patients with an equivalent sore throat should receive equivalent MSEs.^5^ For some conditions, an MSE can be completed within seconds, while for others it cannot be completed within the ED stay, necessitating admission. The CMS notes the MSE “is an ongoing process that begins, but typically does not end, with triage.”^6^ Allegations of violations under EMTALA’s medical screening requirement are numerous.

### Failure to Provide a Medical Screening Exam

One way in which hospitals and physicians are held responsible for violating EMTALA is by not offering an MSE at all. This most frequently arises in situations with patients for whom specialty care is considered more appropriate, in patients exhibiting difficult behavior, and with patients who do not make it to the formal waiting room but still seek ED care. For example, an Ohio ED was reported to the Office of the Inspector General (OIG) for an EMTALA violation after a triage nurse suggested that a pregnant patient in the waiting room seek care at a neighboring hospital with OB services rather than providing an appropriate MSE for her pelvic pain, loss of fluid, and vomiting. Her partner drove her to a facility 30 miles away, where she required an emergency Caesarean section, and her baby was stillborn.^7^

In South Carolina, an ED was fined for an EMTALA violation after a patient brought in after he was assaulted became combative on arrival; security in the ED waiting room told his mother that they would call police if she did not take him out of the ED, and he never received an MSE.^8^ Finally, in Nebraska, an ED entered into a settlement agreement with the OIG after its staff ignored the pleas of a patient and his friend seeking emergency care just outside the ED entrance, refusing to assist the patient out of the car and into the ED. Bystanders eventually helped the patient inside, where he subsequently died from a heart attack less than an hour later.^9^ In each of these cases, a standard MSE is required, and failure to provide one to any patient presenting to the ED may result in penalties. Patients’ difficult behaviors, lack of relevant specialty coverage, or inability to make it to the formal triage desk are not valid justifications for failing to provide an MSE.

### Timely Medical Screening Exam

A second allegation arising from patients in waiting rooms under EMTALA is an inappropriate delay in screening. While EMTALA does not provide specific timelines for provision of an MSE, it does require that the exam adequately reflect the acuity of the patient’s symptoms. For example, in a 2021 Florida case, a patient died in the waiting room from complications of COVID-19 after being unassessed for 10 hours.^10^ In a 2019 Maryland case, a patient who was brought in by paramedics for nausea and vomiting was placed in the hallway to await triage and had three separate seizures over the next 45 minutes before receiving any examination by medical personnel. After his third seizure, he suffered a respiratory arrest and could not be resuscitated.^11^ Both cases led to allegations of EMTALA violations due to delayed screening and resulted in settlements with the OIG.

### Appropriate Medical Screening Exam

A third common allegation under EMTALA is failure to perform an *appropriate* MSE, often highlighted by a departure from the ED’s own policies and procedures. Medical screening exams are considered processes and not just one-time events; therefore, allegations of delays may occur not just at the initial evaluation but also for re-evaluations. For example, in a case settled with a Florida ED, a man initially presented with dysphagia and underwent a computed tomography of the neck that was reassuring. About nine hours later, while still in the waiting room, he developed chest pain, but when he communicated this to the triage staff, no further tests were ordered other than a blood pressure check. He subsequently died in the waiting room due to a ruptured thoracic aortic aneurysm, and the ED was found to have fallen short in its duty to provide an appropriate MSE in response to his concerns of chest pain.^12^ In this case, the change in symptoms necessitated a repeat MSE; the initial MSE for the patient’s previous symptoms was not sufficient to meet EMTALA’s requirement for an “appropriate” MSE when he developed additional symptoms.

The EMTALA does not specify the components of an MSE but instead gauges the exam’s appropriateness based on 1) a determination that it was designed to identify an emergency medical condition and 2) a finding that it is uniformly applied to all patients who present to the ED with similar symptoms or conditions.^5^ Often the MSEs come from the hospital’s internal policies and clinical practice guidelines; these can be a double-edged sword by helping emergency clinicians make quick decisions regarding patient assessments and plans of care, while also creating legal risks when the guidelines are not applied uniformly. Therefore, it is imperative that ED personnel are well informed on the policies and guidelines that the hospital in which they practice has adopted. In situations where a hospital does not have established policies, the applicable professional standard of care takes its place.

## LIABILITY RISKS UNDER NEGLIGENCE ALLEGATIONS

Hospitals and physicians may also face legal risks for management of waiting room patients under general principles of negligence. That is, patients and families may allege that the hospital and physicians failed to meet the standard of care due to the way patients were triaged, screened, treated, or re-evaluated while awaiting definitive care. The allegations at issue may be similar to those in EMTALA cases, but the lawsuits can amount to much larger settlements and verdicts as they are not statutorily limited, as is the case with EMTALA claims.

For example, in a 2013 Pennsylvania case, a 56-year-old man presented to the ED with chest pain and difficulty breathing. Triage staff obtained vitals and an electrocardiogram (ECG), which was interpreted as abnormal. About 35 minutes later, the patient’s family alerted the triage staff that the patient’s pain was worsening, but no additional evaluation was performed. About half an hour later, the patient collapsed in the waiting room and could not be resuscitated. The family sued the physician who read the ECG and the ED group, arguing that the patient was not appropriately triaged or treated. Ultimately, the case was settled for $1.4 million.^13^

In a second case, a two-year-old female was brought to the ED by her parents with fever, rapidly spreading rash, and weakness. She was triaged and directed to the waiting room. Over the course of the next five hours, her parents requested additional evaluations as her rash spread and she continued to worsen. The parents eventually pushed past waiting room personnel into the main ED, where the patient was found to be in septic shock, requiring amputations on all four extremities. Her family sued the hospital and physicians, ultimately settling for $10 million, including the maximum allowed by the physician’s malpractice policy limits.^14^

In these cases, patients may bring these allegations against the hospital and any physicians involved in the MSE, essentially alleging that these parties did not meet their standard of care in some way. In many cases, it is unclear whether the emergency physicians have established a relationship with the patient in the waiting room and are vulnerable to this type of lawsuit. A physician-patient relationship is not legally established until a physician takes an affirmative act on behalf of the patient, which may be as simple as ordering or interpreting their waiting room tests. How the courts will view which actions constitute establishment of this physician-patient relationship is not always predictable. The safest assumption is that *patients in the waiting room are the responsibility of the physicians in the ED*.

## CONCLUSION

With the continuously growing challenges of ED boarding and long wait times, it is imperative that hospitals understand their legal responsibilities to patients in the ED waiting room. The Emergency Medical Treatment and Labor Act requires that all patients who present to the ED receive a timely medical screening exam that is consistently administered for all patients with similar symptoms and conditions. Emergency department staff should routinely document these screenings while a patient is in the waiting room as part of the ongoing MSE process. Since appropriate MSEs are determined by each hospital’s written policies, ED staff—including clinicians, nursing, and administration—must be aware of their hospital’s relevant clinical practice guidelines. When such guidelines are unavailable, they must be aware of the applicable professional standard of care. Emergency clinicians should understand that they may be held responsible for the care provided, or not provided, to patients in the waiting room, even with very little involvement with those patients and no face-to-face time.
